# Consumer Acceptance Comparison Between Seasoned and Unseasoned Vegetables

**DOI:** 10.1111/1750-3841.14027

**Published:** 2018-01-16

**Authors:** Yiming Feng, Marta Albiol Tapia, Kyle Okada, Nuria Blanca Castaneda Lazo, Karen Chapman‐Novakofski, Carter Phillips, Soo‐Yeun Lee

**Affiliations:** ^1^ Univ. of Illinois at Urbana‐Champaign 905 S. Goodwin Ave. Urbana IL 61801 U.S.A

**Keywords:** consumer acceptance, seasoning, vegetables

## Abstract

**Abstract:**

Recent findings show that approximately 87% of the U.S. population fail to meet the vegetable intake recommendations, with unpleasant taste of vegetables being listed as the primary reason for this shortfall. In this study, spice and herb seasoning was used to enhance palatability of vegetables, in order to increase consumer acceptance. In total, 749 panelists were screened and recruited as specific vegetable likers of the vegetable being tested or general vegetable likers. Four sessions were designed to evaluate the effect of seasoning within each type of vegetable, including broccoli, cauliflower, carrot, and green bean. Each panelist was only allowed to participate in one test session to evaluate only one vegetable type, so as to mitigate potential learning effect. Overall, the results showed that seasoned vegetables were significantly preferred over unseasoned vegetables (*P* < 0.001), indicating the sensory properties were significantly improved with seasoning. When general vegetable likers and specific vegetable likers were compared in terms of their preference between seasoned and unseasoned vegetables, the pattern varied across different vegetables; however, general trend of seasoned vegetable being preferred remained. The findings from this study demonstrate the effect of seasoning in enhancing consumer liking of vegetables, which may lead to increased consumption to be assessed in future studies.

**Practical Application:**

To improve the sensory properties of vegetables, masking the bitter taste of vegetables using spice and herb seasoning are gaining increasing attention. Our findings suggest that the overall liking of vegetables could be improved by incorporating spice and herb seasonings that are specifically formulated for each vegetable. Ultimately, developing and commercializing spice and herb seasonings may aid to increase vegetable consumption, as well as expanding the vegetable seasoning market.

## Introduction

Fruits and vegetables are essential components of a healthy diet, as important sources for vitamins, dietary fibers, and other bioactive compounds. In addition to providing daily required nutrients, numerous studies have found that sufficient fruit and vegetable intake helps to reduce the risk of many diseases, such as cancer (Van Duyn and Pivonka 2000; Sato and others 2005),  stroke (Dauchet and others 2005; He and others 2006), and cardiovascular diseases (Bazzano and others 2003).

In 1990, the Dietary Guidelines Committee recommended 3 or more servings of vegetables and 2 or more servings of fruits each day. In addition, a national 5‐A‐Day for Better Health Program was initiated, in order to encourage people to increase their daily consumption of fruits and vegetables (Serdula and others 2004). According to the most recent 2015 to 2020 Dietary Guidelines for Americans, half of the plate should be filled with fruits and vegetables (Agriculture 2015). However, during 2007 to 2010, half of the total U.S. population consumed less than 1 cup of fruit and less than 1.5 cups of vegetables daily (Moore and Thompson 2015), and 87% of the total population were not able to meet vegetable intake recommendations (NSDUH 2014). As such, many efforts have been taken to increase vegetable consumption. To date, most studies have focused intervention strategies on children and young adults (Ahearn 2003; Lowe and others 2004; Blanchette and Brug 2005; Richards and others 2006), and only a few studies were conducted with adults (Rolls and others 2010).

Strong bitterness has been identified as the main cause of the low palatability issues of vegetables, by suppressing endogenous sweetness and enhancing disliked vegetable aromas (Dinehart and others 2006; Sharafi and others 2013). In a recent study by Poelman and others (2017), the authors concluded that vegetables lack in attributes that are innately liked such as sweet and savory and possess innately disliked attributes such as bitter. A substantial portion of these unpleasant tastes and aro are attributed to dietary phytonutrients such as naringin (bitter flavonoid). Despite the bitterness of these compounds, phytonutrients, and their metabolites have been found to act as antioxidants, phytoestrogens, and enzyme inducers (resulting in anticarcinogenic action in the case of some plant flavonoids; Drewnowski and Gomez‐Carneros 2000). Hence, instead of removing phytonutrients, masking is a better option to improve the flavor profiles of vegetables while maintaining their nutritional value (Sharafi and others 2013). Pioneering studies have implied some potentials. One representative study found that salt is capable of masking bitterness (Breslin and Beauchamp 1997). However, it is important to control the sodium amount in seasonings. According to the literature, 99% of U.S. adults consume excess sodium, which may be a factor in 65% of U.S. adults being hypertensive or prehypertensive (Kuo and Lee 2014). Therefore, vegetable seasonings with less sodium content are desired to minimize the adverse effects. Likewise, other studies have proposed the strategy of using spices and herbs to improve the sensory properties of vegetables (Nikolaus and others 2017; Manero and others 2017), yet no sensory consumer tests have been published to provide supporting evidence.

Previous studies treated vegetables as a general terminology (Pollard and others 2002; Schonhof and others 2004). However, each type of vegetable possesses its unique sensory properties, which further leads to different perceptions by each individual consumer. For example, the bitter taste in broccoli is attributed to glucosinolates (Schonhof and others 2004), whereas in cauliflower, allyl isothiocyanate, dimethyl trisulfide, dimethyl sulfide, and methanethiol are the key odorants responsible for rejection by consumers (Engel and others 2002). In some cases, texture profiles would be regarded as a predominant factor in consumer liking of the specific vegetable (Szczesniak and Kahn 1971; Szczesniak 1972). Therefore, for each vegetable, specific and appropriate strategies should be implemented and assessed independently. However, the manifold strategies could also lead to diverged consequences towards different consumer segments. For instance, when a flavor masking strategy is applied to modify sensory properties, those consumers who like the original vegetable flavors could have lower acceptability when the original vegetable flavor is masked. Therefore, it is necessary to comprehensively evaluate the impact of flavor modification on different segments of consumers. According to literature, over 90% of the population consumes vegetables, but in insufficient amounts (Billson and others 1999; Sargeant and others 2001). Therefore, it would be reasonable to hypothesize that these vegetable consumers, who do not eat adequate amounts of vegetables, could increase their consumption if the palatability could be improved. In this study, consumers were screened and recruited in 2 groups, specific vegetable likers of the specific vegetable being tested and general vegetable likers, in order to compare the changes in acceptability of the 2 consumer segments with the flavor modifying treatment of each vegetable.

Utilizing seasonings was regarded as an effective strategy to improve the palatability of vegetables (Li and others 2015). To date, there is still a lack of systematic studies that reflect the effects of seasonings, particularly by means of consumer tests with demographically diverse panelists. Herein, we report a study with the objective of investigating consumers’ acceptance of 4 types of vegetables seasoned with and without seasoning. It was hypothesized that the vegetables seasoned with herbs and spices would receive higher consumer acceptance ratings. It was further hypothesized that the general vegetable likers group will demonstrate a higher increase in liking with seasoning of vegetables than the specific vegetable likers group, because the general vegetable likers group's baseline acceptance rating for the specific vegetable unseasoned would be lower than the specific vegetable likers group; thus, would have more capacity to increase their liking.

## Materials and Methods

### Experimental design

The panelists for this study were recruited mainly by e‐mail, Internet posts, and flyers, and included mostly University students, faculty, staff, and members of the Champaign‐Urbana community. The requirements for participation in the test included being 18 y of age or older, liking vegetables, having no known food allergies and not participating in other portions of the same study. In addition, in order to not create bias for the customers of Bevier Café (Café located in the Food Science building on campus) who may participate in the latter part of the real‐setting study, consumers of vegetables were recruited from students, staff and local community adults who did not eat frequently at Bevier Café, defined as “no more than once every 2 wk” on average. During the recruitment process, the participants were divided into 2 screening groups: panelists in Screening Group A were asked whether they liked consuming vegetables, and only those who defined themselves as vegetable likers were eligible to participate. Panelists in Screening Group B were asked whether they were likers of the particular vegetable being tested, and the same criteria were applied. In total, 754 panelists were recruited in this study, with 389 in Screening Group A and 365 in Screening Group B. Each panelist received a $10 compensation for their participation in the study, and experimental protocol was reviewed and approved by the Institutional Review Board.

### Ingredients

Broccoli, cauliflower, carrots, and green beans were obtained from local grocery stores and were held frozen at −20 °C until use. The ingredients included in the possible seasonings of the different vegetables were: soybean vegetable oil (Harvest Value, U.S. Foods, INC., Rosemont, Ill., U.S.A.), iodized table salt (Morton Salt, Inc., Chicago, Ill., U.S.A.), ground ginger, garlic powder, dried ground cayenne chili pepper, onion powder, dried dill weed, ground black pepper, ground coriander seed, and dried parsley (McCormick & Co, Inc., Hunt Valley, Md., U.S.A). The details about the ingredients are listed in Table 1. The seasoning used for each vegetable was different, because each vegetable has different flavor profile. In previous studies, flavor masking was reported primarily due to the presence of sulfur flavor compounds such as diallyl disulfide (Yang and others 2011). However, the unpleasant flavors present in each vegetable vary widely, so the extent of masking is a result of complex interactions, remaining unpredictable and unexplored (Wagh and Ghadlinge 2009). Therefore, the strategy of developing a particular seasoning in this study was to combine a group of herbs that are rich in sulfur favor compounds, such as onion, pepper, garlic, which levels were optimized based on internal panel discussion.

**Table 1:** List of ingredients for each recipe tested

Product	Amount (g)	Ingredient	Manufacturer
Seasoned broccoli	907 14 3 0.42 0.27 0.42 0.27	Frozen broccoli Pure vegetable soybean oil Iodized table salt Garlic powder Onion powder Dried dill weed Ground black pepper	Monogram, U.S. Foods, Inc. Harvest Value, U.S. Foods, Inc. Morton Salt, Inc. McCormick & Company, Inc. McCormick & Company, Inc. McCormick & Company, Inc. McCormick & Company, Inc.
Unseasoned broccoli	907 14 3	Frozen broccoli Pure vegetable soybean oil Iodized table salt	Monogram, U.S. Foods, Inc. Harvest Value, U.S. Foods, Inc. Morton Salt, Inc.
Seasoned cauliflower	907 14 3 0.8 0.6 0.27 0.8	Frozen cauliflower Pure vegetable soybean oil Iodized table salt Garlic powder Onion powder Ground black pepper Ground coriander seed	Harvest Value, U.S. Foods, Inc. Harvest Value, U.S. Foods, Inc. Morton Salt, Inc. McCormick & Company, Inc. McCormick & Company, Inc. McCormick & Company, Inc. McCormick & Company, Inc.
Unseasoned cauliflower	907 14 3	Frozen cauliflower Pure vegetable soybean oil Iodized table salt	Harvest Value, U.S. Foods, Inc. Harvest Value, U.S. Foods, Inc. Morton Salt, Inc.
Seasoned carrots	907 14 1.5 0.5 0.5 0.15	Frozen carrots Pure vegetable soybean oil Iodized table salt Ground ginger Garlic powder Dried ground cayenne chili pepper	Monogram, U.S. Foods, Inc. Harvest Value, U.S. Foods, Inc. Morton Salt, Inc. McCormick & Company, Inc. McCormick & Company, Inc. McCormick & Company, Inc.
Unseasoned carrots	907 14 1.5	Frozen carrots Pure vegetable soybean oil Iodized table salt	Monogram, U.S. Foods, Inc. Harvest Value, U.S. Foods, Inc. Morton Salt, Inc.
Seasoned green beans	907 9 3 0.42 0.27 0.42 0.27	Frozen green beans Pure vegetable soybean oil Iodized salt Garlic powder Onion powder Dried parsley herb Ground black pepper	Monogram, U.S. Foods, Inc. Harvest Value, U.S. Foods, Inc. Morton Salt, Inc. McCormick & Company, Inc. McCormick & Company, Inc. McCormick & Company, Inc. McCormick & Company, Inc.
Unseasoned green beans	907 9 3	Frozen green beans Pure vegetable soybean oil Iodized salt	Monogram, U.S. Foods, Inc. Harvest Value, U.S. Foods, Inc. Morton Salt, Inc.

### Sample preparation

The vegetables were cooked in the kitchen facility of Bevier Café, following a pre‐designed protocol. Precisely, each batch containing 907.2 g of vegetables were steamed in a perforated pan in an oven for 5 min, and then tossed with pre‐weighed soybean oil and salt. This is a typical preparation for vegetable (Poelman and others 2015), and has been used in previous research design (Manero and others 2017). If no further seasoning was used besides oil and salt, the samples were designated as “unseasoned,” because it is the herbs and spices that are the treatment of interest in the study. The samples designated “seasoned” were added a premeasured seasoning blend in addition to the oil and salt. Both seasoned and unseasoned vegetable samples were coded with randomized 3‐digit number codes to avoid bias.

As for sample presentation, each panelist was served a tray that included the 2 coded samples (in 162.7 mL cups with lids), a paper ballot, a pen, a napkin, 3 water cups according to the rinse protocol which was carbonated water, followed by warm water, and room temperature water, and an expectorating cup with lid.

### Sensory evaluation procedure

The sensory evaluation sessions took place in a large restaurant‐style room on the Univ. of Illinois at Urbana‐Champaign campus (Bevier Hall Spice Box) under incandescent lighting. Two versions of the scorecard were made for each screening group for the purposes of randomizing the order of sample presentation. These were distributed randomly to the panelists to avoid bias.

Panelists 1st evaluated both samples for overall acceptance on a 9‐point hedonic scale (Lawless and Heymann 1999), anchored with 1 = “dislike extremely,” 5 = “neither like nor dislike,” and 9 = “like extremely.” After overall acceptance, panelists rated each sample on the 9‐point hedonic scale for appearance and flavor attributes, and were asked a series of optional open questions that included what they liked about the sample, what they disliked, and recommendations based on their own criteria to improve the sample. Open‐ended questions were not asked in the green bean test, which was conducted in the 1st round of the vegetable acceptance tests. The other 3 vegetables were tested in the second round with open‐ended questions. Other questions regarding demographics and vegetable consumption behavior were asked following the acceptance questions.

Before tasting each sample, the panelists were instructed to rinse their mouth with carbonated water, warm water, and room temperature water. Carbonated water, warm water, and room temperature water are all commonly used rinses for sensory studies. For different types of test samples, rinsing protocol vary. Carbonated water is widely used to rinse oily samples. Warm water is used when samples are served with elevated or lowered temperatures such as ice creams (Lucak 2008). In terms of vegetables, there is very limited information about the palate cleansing protocols in the literature. Considering that the samples were served at elevated temperature and also contained some oil, 3 rinses were considered appropriate to sufficiently cleanse the palate: carbonated water, warm water, and room temperature water. The rinsing protocol was developed according to preliminary test, which was able to sufficiently remove the prior taste residual. The panelist could expectorate the samples after evaluation expectorating the rinses as well or swallow the samples in which case they were asked to swallow the rinses as well.

Data were collected on paper ballots and entered into Microsoft Excel spreadsheets to be analyzed. The ballots were coded to be anonymous and were all treated confidentially by the investigators of the study.

### Statistical analysis

Data were analyzed based on 754 ballots, using the paired *t*‐test method in Microsoft Excel (Microsoft 2013, Redmond, Wash., U.S.A.) to compare results between samples within each screening or demographic group. The significance level was set at 0.05. For agglomerative cluster analysis and correspondence analysis, XLSTAT 2014.4.09 (Addinsoft, New York, N.Y., U.S.A.) was used. Agglomerative cluster analysis was conducted following a methodology described in a previous study (Koga and others 2016). Panelists were grouped according to their overall liking scores of samples for broccoli, cauliflower, carrots, and green beans, respectively. For the verbal comments, keywords frequency was analyzed using Nvivo (Nvivo 12, QSR Intl. Inc., Burlington, Mass., U.S.A.).

## Results and Discussion

### Overall liking combined

Table 2 shows the average overall consumers’ acceptance between seasoned and unseasoned vegetables inclusive of all types of vegetables. Across all the testing groups, general vegetable likers, specific vegetable likers, and combined consumers, paired *t*‐tests consistently showed that seasoned vegetables are significantly liked more by consumers. This significant finding suggests that sensory properties of vegetables may have been improved by spices and herbs, through masking and modifying the often bitter taste of vegetables. It is also interesting to note that the vegetables tested in this study were well‐liked by the consumers demonstrated by the average overall liking scores in the range of 6.72 to 7.00. Considering the most updated sodium adequate intake (AI, 1500 mg) and upper intake level (UL, 2300 mg) for young adults (Kuo and Lee 2014), consumption of 3 vegetable portions with seasonings would contribute to 24% and 16% of the daily recommended intake, respectively of AI and UL, which are acceptable.

**Table 2:** Average overall liking scores^a^ across all vegetables

	General vegetable likers (*n* = 385)		Specific vegetable likers (*n* = 364)		Combined (*n* = 749)	
	Seasoned	Unseasoned	Seasoned	Unseasoned	Seasoned	Unseasoned
Average acceptance	6.96 ± 1.61	6.74 ± 1.54	7.00 ± 1.61	6.72 ± 1.56	6.98 ± 1.61	6.73 ± 1.55
*P*‐value^b^	0.032		0.004		0.0005	

a On a 9‐point hedonic scale, anchored 9 = like extremely, 5 = neither like nor dislike, and 1 = dislike extremely.

b Probability based on paired *t*‐test.

### Overall liking for each vegetable

Consumer acceptance, along with attribute acceptability ratings of flavor and appearance of seasoned and unseasoned vegetables within each test session is shown in Table 3. For broccoli, seasoning was found to significantly improve the overall liking for specific broccoli likers while not making significant difference for the general vegetable likers. Broccoli has been reported for its health benefits (Jeffery and others 2003; Jeffery and Araya 2009), which might have led to the recent increased consumption (Hayley Boriss and Henrich Brunke 2005). Due to the unpleasant taste attributed by glucosinolates (Schonhof and others 2004), many culinary techniques have been used to produce palatable broccoli products. Therefore, broccoli likers might possess rather higher expectations of broccoli than general vegetable likers. As a result, a greater discrepancy between seasoned and unseasoned broccoli was displayed in specific broccoli likers. The results also imply that broccoli likers are more sensitive to herb and spice seasonings, compared to general vegetable likers.

**Table 3:** Average overall liking scores^a^ from a consumer panel comparing seasoned and unseasoned vegetables within each vegetable group

Vegetable	Consumer group	Seasoned vs. unseasoned	Overall liking	Flavor liking	Appearance liking
Broccoli	Specific likers	Seasoned	7.40**	7.34*	6.81
		Unseasoned	6.90**	6.80*	6.82
		*P*‐value^b^ (*n* = 96)	0.006	0.010	0.951
	General likers	Seasoned	7.13	7.23	6.67*
		Unseasoned	7.32	7.27	7.04*
		*P*‐value (*n* = 98)	0.31	0.849	0.032
Cauliflower	Specific likers	Seasoned	7.22	7.18	6.81**
		Unseasoned	7.12	6.96	6.31**
		*P*‐value (*n* = 91)	0.638	0.351	0.009
	General likers	Seasoned	7.13*	7.03	6.71**
		Unseasoned	6.70*	6.64	5.99**
		*P*‐value (*n* = 103)	0.016	0.055	0.001
Carrots	Specific likers	Seasoned	6.25	6.16	5.92***
		Unseasoned	6.16	6.07	6.54***
		*P*‐value (*n* = 94)	0.650	0.735	0.0002
	General likers	Seasoned	6.61	6.54	6.18**
		Unseasoned	6.24	6.15	6.64**
		*P*‐value (*n* = 102)	0.089	0.127	0.003
Green beans	Specific likers	Seasoned	7.13*	7.27**	6.75
		Unseasoned	6.70*	6.63**	6.73
		*P*‐value (*n* = 83)	0.011	0.0063	0.958
	General likers	Seasoned	6.98	7.09	6.41
		Unseasoned	6.74	6.59	6.83
		*P*‐value (*n* = 82)	0.313	0.056	0.0576

a On a 9‐point hedonic scale, anchored 9 = like extremely, 5 = neither like nor dislike, and 1 = dislike extremely.

b Probability based on paired *t*‐test comparison of seasoned and unseasoned samples.

^*^ *P* < 0.05.

^**^ *P* < 0.01.

^***^ *P* < 0.001.

For cauliflower, the overall liking was significantly increased by seasoning among general vegetable likers but showed no difference in specific cauliflower likers. Different from broccoli, the yearly consumption and yield of cauliflower has dropped recently over time. According to the 2015 data (USDA‐NASS 2016), the production of cauliflower dropped to 2400 tons compared to broccoli at 39200 tons. Although the population size of cauliflower consumers is relatively small, those general vegetable likers were found to rate seasoned cauliflower significantly higher, which suggests a great potential of increasing the number of cauliflower consumers by incorporating herb and spice seasonings into dishes made with cauliflower.

No significant difference was found between seasoned and unseasoned carrots, neither in the specific carrot likers group nor general vegetable likers group. This may be due to masking of natural carrot sweetness by seasoning, which can be supported by the open comment data from keywords frequency analysis which is further discussed in verbal information analysis. Another possible reason may be the type of seasoning for carrots that was used, which imparted spiciness because it included cayenne chili pepper. Spicy food is not common to all cultures or traditional cuisines (Ludy and Mattes 2012), although commonly found in vegetable seasonings (Nikolaus and others 2017), so that might have caused a decrease in the scores given to the seasoned carrots. Disliking of the spiciness was mentioned by various panelists in the open comment section of the questionnaire.

For green beans, a significant difference between seasoned and unseasoned samples was found in the specific likers group but not in the general vegetable likers group. In green beans, linoleic, and linolenic acids were reported as principal volatile compounds (Lumen and others 1978). However, the presence of linoleic acids suppresses the sensitivity of saltiness perception, by increasing taste thresholds (Mattes 2006). By adding spice and herb seasonings, a more complicated flavor interaction could occur between seasonings and original green bean flavors, causing synergistic perceptual experience and hence increasing acceptability for the specific vegetable likers group.

### Specific attributes

For all test sessions, flavor and appearance liking were the 2 attributes rated by consumers, in order to potentially identify the drivers of liking or disliking. Data in Table 3 illustrates that flavor plays a more significant role in the overall liking than appearance. Two data sets of flavor results showed significant difference between seasoned and unseasoned samples, and both of them led to significant difference in overall liking. However, only one out of 5 significant appearance liking differences resulted in overall liking difference. Flavor is more likely to be the driver of liking for vegetables, which has been well established in a previous study (Cox and others 2012). Similar results were also found in other studies with sweet potato and orange juice, that suggest that flavor components are more likely to be drivers of liking in the vegetables being tested (Leksrisompong and others 2012; Kim and others 2013).

### Ethnicity differences

Data regarding overall liking of all the tested vegetables were combined for a better overview of the general preferences across different ethnic groups. Analysis of the data revealed that both Asians and Caucasians liked the seasoned vegetables significantly more than the unseasoned vegetables (*P* < 0.05). The combined percentage of these 2 ethnic groups together in this study was more than 85%.

### Cluster analysis

**Figure 1:**
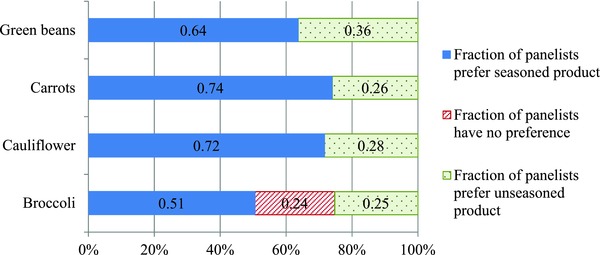
Distribution of panelists on their preference of seasoned or unseasoned vegetables.

Panelists were clustered according to their overall liking ratings across all samples, and the results are shown in Table 4. Three clusters were identified for all tests based on their acceptance pattern. Overall, these clusters can be classified into 3 categories: panelists who rated seasoned vegetables significantly higher, panelists who rated unseasoned vegetables significantly higher, and panelists who did not prefer one over the other. In terms of 4 vegetables tested in this study, the seasoned samples were rated significantly higher in all the largest clusters identified as Cluster 1 for each vegetable other than green beans demonstrating that majority of consumers preferred the seasoned samples. In green beans, the largest cluster (Cluster 1) show higher liking for unseasoned sample; however, the other slightly smaller clusters (Clusters 2 and 3) both show significantly higher liking in seasoned samples; thus, combined, they would comprise a larger cluster than Cluster 1, still demonstrating that the majority of consumers for green beans significantly prefer seasoned sample. Figure 1 further illustrates these 3 preference patterns. The fraction of panelists who preferred seasoned vegetables is greater than the fraction of panelists who preferred unseasoned vegetables and the fraction of panelists who did not have preference, which demonstrates that seasoning was able to improve the sensory properties of vegetables for the majority of consumers.

**Table 4:** Average overall liking^a^ of samples for each cluster of panelists for different vegetables

Broccoli		Cluster 1 (*n* = 98)	Cluster 2 (*n* = 49)	Cluster 3 (*n* = 47)
	Seasoned	7.36^b^, ***	5.84***	8.55
	Unseasoned	6.03***	7.96***	8.47
		Cluster 1 (*N* = 97)	Cluster 2 (*N* = 55)	Cluster 3 (*N* = 42)
Cauliflower	Seasoned	8.30***	5.58***	6.64***
	Unseasoned	7.53***	7.38***	4.81***
		Cluster 1 (*N* = 117)	Cluster 2 (*N* = 51)	Cluster 3 (*N* = 28)
Carrots	Seasoned	7.53 ***	3.98***	6.32***
	Unseasoned	7.01***	5.90***	3.36***
		Cluster 1 (*N* = 60)	Cluster 2 (*N* = 56)	Cluster 3 (*N* = 49)
Green beans	Seasoned	5.78***	8.48***	6.98***
	Unseasoned	7.27***	7.66***	4.98***

a On a 9‐point hedonic scale, anchored 9 = like extremely, 5 = neither like nor dislike, and 1 = dislike extremely.

b Significance based on paired *t*‐test comparison of seasoned and unseasoned samples.

^*^ *P* < 0.05.

^**^ *P* < 0.01.

^***^ *P* < 0.001.

### Verbal information analysis

The verbal analysis results for the open questions in the ballot are shown in Table 5. It is based on the rule‐of‐thumb that the frequency of use of certain words is related to their importance (Guerrero and others 2000). Raw data collected were further processed by merging similar and stemmed words, and only those words that were relevant to sensory perceptions were counted. The panelists were free to use the words they considered appropriate to describe the samples, and there were no keys or suggestions provided. The words “flavor” and “taste” might have been used indistinctively by consumers, but were not grouped together to maintain the accuracy of the responses. Table 5 illustrates the top 5 keywords used to describe the reasons of liking or disliking each sample, in which case these 5 keywords could be preliminarily identified as the drivers of liking or disliking.

**Table 5:** Frequency of keywords being used in consumers’ open comments

Reasons for liking				Reasons for disliking			
General likers		Specific likers		General likers		Specific likers	
Seasoned Broccoli							
Flavor	18.02%	Flavor	13.27%	Taste	9.87%	flavor	8.79%
Tastes	6.76%	Taste	6.80%	Flavor	7.89%	Taste	4.40%
Color	5.41%	Seasoning	6.47%	Looks	3.95%	Looks	3.30%
Seasoning	4.95%	Texture	4.53%	Mushy	3.95%	Soft	3.30%
Soft	3.60%	Looks	2.91%	Soft	3.29%	Appearance	2.75%
Unseasoned Broccoli							
Flavor	11.16%	Flavor	8.46%	Flavor	15.18%	Taste	7.73%
Tastes	9.16%	Tastes	8.46%	Bland	6.25%	Flavor	6.82%
Texture	6.37%	Fresh	6.25%	Taste	6.25%	Bland	5.45%
Fresh	5.58%	Texture	6.25%	Seasoning	4.46%	Salty	4.55%
Cooked	3.98%	Color	4.78%	Salty	3.57%	Seasoning	3.64%
Seasoned cauliflower							
Flavor	16.80%	Flavor	12.20%	Seasoning	12.90%	Flavor	6.82%
Texture	7.81%	Taste	9.35%	Flavor	10.48%	Taste	6.06%
Seasoning	7.42%	Seasoning	8.54%	Spices	4.84%	Seasoning	4.55%
Taste	6.25%	Spices	5.28%	Appearance	4.03%	Cauliflower	3.79%
Spices	4.69%	Look	4.07%	Salty	4.03%	Salty	3.79%
Unseasoned cauliflower							
Flavor	9.73%	Flavor	9.92%	Bland	15.08%	Bland	11.02%
Taste	7.96%	Taste	7.44%	Plain	7.14%	Flavor	11.02%
Texture	7.08%	Texture	5.37%	Flavor	6.35%	Plain	8.47%
Fresh	5.75%	Cooked	4.96%	Look	6.35%	Look	3.39%
Buttery	4.42%	Fresh	3.72%	Boring	5.56%	Seasoned	3.39%
Seasoned carrots							
Flavor	16.09%	Flavor	15.46%	Flavor	6.35%	Flavor	8.25%
Taste	9.57%	Taste	10.31%	Taste	6.35%	Soft	5.67%
Sweet	5.22%	Sweetness	6.19%	Texture	5.82%	Taste	5.67%
Seasoning	4.78%	Soft	4.64%	Spicy	5.29%	Smell	4.64%
Texture	3.91%	Color	4.12%	Look	4.76%	Spicy	4.64%
Unseasoned carrots							
Sweetness	10.27%	Sweet	11.11%	Flavor	10.62%	Flavor	9.93%
Taste	9.38%	Taste	10.65%	Texture	6.25%	Taste	9.27%
Color	6.25%	Flavor	8.80%	Soft	5.00%	Texture	7.28%
Flavor	6.25%	Carrot	5.09%	Taste	5.00%	Soft	6.62%
Appearance	4.91%	Soft	4.17%	Carrots	4.38%	Bland	4.64%

For broccoli, general vegetable likers and specific broccoli likers used almost the same keywords to claim their reasons of liking or disliking. Flavor and taste ranked the most frequent words in the comments, indicating their predominating influence in determining consumer acceptance. Other words related to appearance and texture were also mentioned many times. When comparing the reasons for liking seasoned or unseasoned broccoli, the use of different words was also noticed. “Seasoning” is mentioned frequently for liking seasoned broccoli while “fresh” was a keyword for liking unseasoned broccoli, which implies their unique drivers of liking.

For the cauliflower data, a very similar pattern to broccoli was found. General vegetable likers and specific cauliflower likers used almost the same words to describe the samples. It is noteworthy that general vegetable likers used the words “bland,” “plain,” and “boring” to describe the taste of unseasoned cauliflower, indicating their very low acceptance to unseasoned cauliflower. This provides an explanation to the significant difference between seasoned and unseasoned cauliflower being found in general likers but not in specific likers, because the unflavored cauliflower was deemed too bland for those consumers who do not particularly like to eat cauliflower.

As for the carrot study, no significant difference between seasoned and unseasoned carrots was found in both general vegetable likes and specific carrot likers. In both general likers and specific likers, sweetness was mentioned as the predominant reason for liking unseasoned carrots. The spicy seasoning may have been incongruent with the sweetness of carrots, which may have resulted in no significant difference in liking between the seasoned and unseasoned. In addition, in a cafeteria purchase study, carrots were purchased less frequently and liked less than broccoli or green beans (Nikolaus and others 2017).

### Questionnaire

The questionnaire results (Table 6) show that broccoli, cauliflower, green beans and carrots are the vegetables that are most frequently consumed with seasonings, which provides the justification for the 4 vegetables tested in this study. The data also suggests that development of vegetable seasoning could be focused on these above‐mentioned vegetables. When consumers were asked about their general seasoning preferences, more people chose salt (83.4%) and pepper (71.1%) over herbs (57.2%) and spices (52.1%) as their frequent seasoning options. Focus group results have also suggested that while salt and pepper are the most commonly used seasonings on vegetables, and that seasonings are thought to enhance flavor, seasoning vegetables was not a strategy that participants offered as suggestions to increase vegetable intake (Nikolaus and others 2017). This study shows that vegetables seasoned with herbs and spices are preferred even though the most common seasonings used by the consumers are salt and pepper. These results imply a potential opportunity in the market to commercialize vegetable seasonings with spices and herbs.

**Table 6:** Demographic information and questionnaire

		Total number		
Characteristic	Category	Screening group A	Screening group B	Total
Age^a^	18–25	281 (37.4%)	248 (33.0%)	529 (70.3%)
	26–35	57 (7.6%)	70 (9.3%)	127 (16.9%)
	36–45	22 (2.9%)	17 (2.3%)	39 (5.2%)
	46–55	24 (3.2%)	17 (2.3%)	41 (5.5%)
	56–65	4 (0.5%)	9 (1.2%)	13 (1.7%)
	>65	1 (0.1%)	2 (0.3%)	3 (0.4%)
Gender^a^	Male	102 (13.6%)	94 (12.6%)	196 (26.2%)
	Female	287 (38.3%)	266 (35.5%)	553 (73.8%)
Ethnicity^b^	American Indian or Alaskan Native	2 (0.3%)	2 (0.3%)	4 (0.5%)
	Asian	186 (23.5%)	136 (17.2%)	322 (40.8%)
	Black or African American	14 (1.8%)	18 (2.3%)	32 (4.1%)
	Caucasian	185 (23.4%)	170 (21.5%)	355 (44.9%)
	Hispanic or Latino	23 (2.9%)	43 (5.4%)	66 (8.4%)
	Native Hawaiian or Other Pacific Islander	0	0	0
Seasoning preference^b^	Salt	327 (84.1%)	300 (82.6%)	627 (83.4%)
	Pepper	272 (69.9%)	263 (72.5%)	535 (71.1%)
	Herb	228 (58.6%)	202 (55.6%)	430 (57.2%)
	Spice	204 (52.4%)	188 (51.8%)	392 (52.1%)
	Other	73 (18.8%)	64 (17.6%)	137 (18.1%)
Seasoned vegetable preference^b^	Broccoli	336 (86.4%)	317 (87.3%)	653 (86.8%)
	Brussel sprouts	214 (55.0%)	190 (52.3%)	404 (53.7%)
	Cabbage	205 (52.7%)	178 (49.0%)	383 (50.9%)
	Carrots	236 (60.7%)	230 (63.4%)	466 (62.0%)
	Cauliflower	283 (72.8%)	266 (73.3%)	549 (73.0%)
	Cucumber	152 (39.1%)	122 (33.6%)	274 (36.4%)
	Lettuce	143 (36.8%)	123 (33.9%)	266 (35.4%)
	Green beans	287 (73.8%)	276 (76.0%)	563 (74.9%)
	Celery	94 (24.2%)	78 (21.5%)	172 (22.9%)
	Other	44 (11.3%)	51 (14.0%)	95 (12.5%)

a Total does not add up to total number of panelists because some panelists decided not to answer this question.

b Total exceeds total number of panelists because more than one option could be chosen.

## Conclusions

The findings from this study demonstrated that seasoned vegetables feature significantly greater acceptance rating compared to unseasoned vegetables. It was also found that the preference of specific and general vegetable likers diverges across different types of vegetables. The reason is attributed to the complexity of sensory properties when vegetables are mixed with seasonings. Future research could further reveal the interaction between the aromatic compounds innate to the vegetables and in different types of seasoning in order to identify the optimal pairing of vegetable and seasoning. It would also be interesting to identify the chemical compounds in seasoning that interact with flavor components of different vegetable types, in order to precisely model how the flavor combinations would come about serving as drivers of liking. A limitation of this study is that keyword frequency analysis lacks accuracy, due to the subjectivity of extracting one single word from a phrase. For future studies, focus groups may be conducted to probe the reasons for liking or disliking a vegetable product.
